# Histone deacetylases in monocyte/macrophage development, activation and metabolism: refining HDAC targets for inflammatory and infectious diseases

**DOI:** 10.1038/cti.2015.46

**Published:** 2016-01-29

**Authors:** Kaustav Das Gupta, Melanie R Shakespear, Abishek Iyer, David P Fairlie, Matthew J Sweet

**Affiliations:** 1 Institute for Molecular Bioscience, The University of Queensland, St Lucia, Queensland, Australia; 2 IMB Centre for Inflammation and Disease Research, The University of Queensland, St Lucia, Queensland, Australia; 3 Australian Infectious Diseases Research Centre, The University of Queensland, St Lucia, Queensland, Australia

## Abstract

Macrophages have central roles in danger detection, inflammation and host defense, and consequently, these cells are intimately linked to most disease processes. Major advances in our understanding of the development and function of macrophages have recently come to light. For example, it is now clear that tissue-resident macrophages can be derived from either blood monocytes or through local proliferation of phagocytes that are originally seeded during embryonic development. Metabolic state has also emerged as a major control point for macrophage activation phenotypes. Herein, we review recent literature linking the histone deacetylase (HDAC) family of enzymes to macrophage development and activation, particularly in relation to these recent developments. There has been considerable interest in potential therapeutic applications for small molecule inhibitors of HDACs (HDACi), not only for cancer, but also for inflammatory and infectious diseases. However, the enormous range of molecular and cellular processes that are controlled by different HDAC enzymes presents a potential stumbling block to clinical development. We therefore present examples of how classical HDACs control macrophage functions, roles of specific HDACs in these processes and approaches for selective targeting of drugs, such as HDACi, to macrophages. Development of selective inhibitors of macrophage-expressed HDACs and/or selective delivery of pan HDACi to macrophages may provide avenues for enhancing efficacy of HDACi in therapeutic applications, while limiting unwanted side effects.

Macrophages are key innate immune cells occupying specific tissue niches throughout the body. The cells express a plethora of receptor systems that allow them to sense the extracellular environment with exquisite sensitivity and specificity. Such receptors include phagocytic receptors, cytokine receptors, receptors for other host-derived inflammatory mediators and several families of danger-sensing pattern recognition receptors, such as the Toll-like receptors (TLRs) and Nod-like receptors. The combined actions of these detection systems enable macrophages to initiate appropriate inflammatory programs upon perturbation of homeostasis. However, dysregulated and/or chronic inflammation driven by macrophages is central to the pathology of many disease states including autoimmune diseases, cardiovascular diseases, neurodegenerative diseases, inflammatory diseases, infectious diseases and cancer.

Through recruitment of inflammatory monocytes and/or local expansion of macrophages at the inflammatory lesion, the host can respond to perturbed homeostasis. Another response mechanism involves changes in macrophage metabolic state, to enable the assignment of available nutrients into distinct metabolic pathways. Indeed, the coordinated control of energy metabolism and cellular activation is essential for innate immune functions.^1^ Whereas quiescent macrophages primarily use oxidative phosphorylation (OXPHOS) to generate energy in the form of ATP,^2^ metabolic pathways in TLR-activated macrophages are skewed from mitochondrial respiration toward aerobic glycolysis.^3^ This switch leads to changes in intracellular levels of metabolites with signaling, antimicrobial and/or immunomodulatory functions. Hence, there has been considerable interest in defining the precise molecular switches that control metabolic processes in macrophages.

Acetyl coenzyme A (acetyl-CoA), which is a central molecule in metabolic pathways,^4^ also acts as a donor molecule for acetylation of lysine residues on proteins. This process is controlled by histone acetyl transferases and histone deacetylases (HDACs), which regulate the acetylation status of histones and thousands of non-histone proteins. The 18 human HDACs are categorized into either classical HDACs or sirtuins, based on their enzymatic mechanisms of action. Classical HDACs (HDAC1-11) share a conserved deacetylase domain that contains a zinc-binding pocket, whereas non-classical HDACs (the sirtuins) act in an NAD^+^-dependent manner. Within the classical HDACs, class I HDACs (HDAC1, 2, 3, 8) can assemble multi-component co-repressor complexes and often act as transcriptional repressors. Nonetheless, they can also promote the expression of specific target genes in some signaling pathways, for example, in type I interferon (IFN) signaling.^5^ Class II HDACs (HDAC4, 5, 6, 7, 9, 10) have both cytoplasmic and nuclear functions, and the class IIa HDACs (HDAC4, 5, 7, 9) can function by both deacetylase-dependent and -independent mechanisms. Collectively, these properties extend HDAC functions well beyond histone deacetylation.

The classical HDACs have essential roles in both the development^6^ and activation^7^ of many immune cell types, including macrophages. Moreover, HDAC inhibitors are efficacious in animal models of several inflammation-related diseases.^8^ Such findings have generated great interest, particularly in relation to the potential development of isoform- or class-specific HDAC inhibitors. Recent reviews have overviewed roles for HDACs in inflammation and immunity.^7, 9^ Given established links between HDACs and macrophages, as well as macrophages and many disease processes, in this review we specifically focus on the involvement of HDACs in regulating macrophage differentiation, activation and metabolism. We also discuss approaches that can be used to selectively target HDACs in macrophages, as these could potentially be harnessed for clinical development.

## Monocyte/macrophage differentiation and regulation by HDACs

### The many faces of macrophage development

The vertebrate hematopoietic system is generated by two highly coordinated, successive waves. In the mouse embryo, ‘primitive' hematopoiesis is a transient early wave occurring from E7 in the yolk sac and giving rise to multi-lineage erythro-myeloid progenitors, which express the Csf-1r.^10, 11^ These cells migrate to the fetal liver before E10.5 and give rise to fetal erythrocytes, monocytes, macrophages and granulocytes. Yolk sac-derived phagocytes have the potential to seed various tissues and to be maintained as resident tissue macrophages throughout adulthood, independently of bone marrow progenitors.^11, 12, 13, 14^ At approximately E10, ‘definitive' hematopoiesis begins with the emergence of hematopoietic stem cells (HSCs) in the aorta–gonadal–mesonephros region.^15^ These cells are characterized by their ability to engraft an irradiated recipient and provide a long-term supply of all blood cell populations throughout adult life. Once the circulatory system begins to function at E10.5, they migrate through the circulation to colonize the fetal liver and placenta. The fetal liver operates as the major hematopoietic organ after E11.5. Here, HSC are expanded before their migration to the thymus (E11-12), spleen (E12) and bone marrow (E16 onward) before birth.

Adult tissue-resident macrophages are maintained by at least two mechanisms. The traditional view is one of a linear process in which committed bone marrow progenitors gave rise to circulating blood monocytes, which, upon entering tissues, differentiate into mature macrophages. HSC are a multipotent, self-renewing population that give rise to multipotent progenitors, which can further commit to distinct differentiation programs. Common myeloid progenitors have the potential to differentiate into the megakaryocyte/erythroid lineage, as well as into granulocytes, dendritic cells (DCs) and monocytes/macrophages. In steady-state adult hematopoiesis, production of myeloid cells is tightly controlled by several growth factors including stem cell factor, interleukin-3 (IL-3), granulocyte-macrophage colony-stimulating factor, granulocyte colony-stimulating factor, colony-stimulating factor 1 and IL-34. CSF-1 is particularly important for monocyte/macrophage development, as mice deficient in this growth factor or its receptor are severely monocytopenic and several tissue-resident macrophage populations are absent or reduced.^16^

The derivation of resident tissue macrophages from monocytes is unequivocally the case for some populations such as those of the colonic mucosa.^17^ However, sophisticated fate-mapping experiments have revealed that tissue macrophages can also be seeded from primitive yolk-sac progenitors before birth, and can be maintained as a self-renewing population independent of bone marrow progenitors. This is particularly the case for microglia in the brain.^18^ However, studies on the contribution of embryonic phagocytes to adult macrophage populations have typically been conducted on young adult mice, and under homeostatic conditions. Under physiological stress, as occurs during infection and/or injury, effector cells are in high demand and must be replenished by emergency myelopoeisis. This is driven by enhanced production of myeloid growth factors such as granulocyte-macrophage colony-stimulating factor and colony-stimulating factor 1,^19^ inducible production of cytokines such as IL-1β and IL-6 that can also direct myelopoiesis, and through direct activation of the HSC themselves.^20^ In these circumstances, monocytes are a major source of the macrophages within the inflammatory lesion. Even during ageing, tissue-resident macrophages in the heart^21^ and lung^11^ are replenished from monocytes, and thus monocytes do indeed make a major contribution to the tissue macrophage population during the lifespan of an animal. Nonetheless, the distinct mechanisms by which tissue macrophage populations can be generated has significant implications for understanding macrophage functions in different microenvironments and disease states.

### HDACs: pulling the strings of HSC to monocyte to macrophage differentiation

Cell fate decisions in the hematopoietic hierarchy are controlled by epigenetic mechanisms. Much of our initial knowledge of HDAC-mediated control of monocyte/macrophage development is derived from clinical trials of HDAC inhibitors as anticancer agents. Pan-HDAC inhibition can cause myelosuppression, particularly thrombocytopenia,^22^ thus implicating HDACs in monocyte/macrophage development. Studies in non-malignant cells also support the view that HDACs regulate myeloid development. For example, treatment of CD34^+ve^ cord blood cells with the HDAC inhibitor (HDACi) valproic acid (VPA) inhibited erythroid differentiation, but promoted myelo-monocytic differentiation.^23^ This was correlated with an increase in PU.1 expression. In contrast, treatment of mature macrophages with HDACi markedly decreased PU.1 protein expression,^24, 25^ implying that HDACs may differentially regulate PU.1 in mature macrophages versus precursors.

Genetic studies also implicate HDACs in HSC fate decisions and myeloid development.^26^ Class I HDAC expression was low in CD34^+ve^ human progenitor cells, increased in more differentiated CD34^−ve^ progenitors and erythroid precursors, but was decreased in myeloid cells. Forced overexpression of HDAC1 by reconstitution of HDAC1-transduced progenitor cells into irradiated donors resulted in reduced CD11b^+ve^ myeloid cells but normal red blood cell numbers 10 weeks after transplantation, suggesting that HDAC1 function must be downregulated during myelopoiesis.^26^ Other reports have identified essential roles for HDAC1, HDAC2 and HDAC3 in the maintenance of homeostatic HSC, as well as committed myeloid progenitor cells.^27, 28^ Interestingly, myeloid-specific deletion of HDAC3 did not interfere with the capacity of bone marrow cells to differentiate to macrophages *in vitro*.^29^ Combined, these studies suggest that HDAC3 is most likely to control very early cell fate decisions during myeloid differentiation. However, gene silencing of HDAC3 in human cord blood stem cells expanded CD34^+ve^ immature progenitors without compromising their ability to self-renew and repopulate the hematopoietic compartment.^30^ In this study, monocyte lineage commitment and maturation of monocyte precursors was not affected, suggesting that HDAC3 controls HSC expansion but not differentiation potential. Whether this relates to differences in experimental systems (for example, knockout versus gene silencing) remains to be determined.

As cells progress down the hematopoietic hierarchy and make increasing commitments to specific cell lineages, other HDACs appear to be important in determining cell fates. For example, HDAC7 repressed the differentiation of pre-B cells to myeloid cells in a transdifferentiation model of lymphoid-primed multipotent progenitor to B-cell commitment choice.^31^ In contrast, HDAC7 is expressed in mature primary mouse macrophages,^32^ the BV2 mouse microglial cell line^33^ and human macrophage-like cell lines.^34^ Collectively, these studies suggest that HDAC7 is transiently downregulated during macrophage differentiation, and then re-expressed to enable it to participate in mature macrophage functions (see ahead). Another classical HDAC implicated in myeloid cell fate decisions is HDAC11. Using transgenic mice expressing green fluorescent protein from the *Hdac11* promoter, Sahakian *et al.*^35^ observed high reporter gene expression in bone marrow granulocytes and low expression in monocytes. Using global *Hdac11* knockout mice, the authors demonstrated that this HDAC negatively regulates myeloid-derived suppressor cell development and function, at least in part through control of suppressive IL-10 production. Induction of emergency myelopoiesis using Freund's adjuvant in *Hdac11*^−/−^ mice resulted in a greater expansion of granulocytes as compared with wild-type mice, although studies with myeloid-specific *Hdac11* knockout mice are necessary to determine cell-intrinsic effects of HDAC11 in regulating myeloid development. Collectively, the above literature suggests that class I HDACs have important roles in maintaining the stem cell pool and directing early lineage choices. As commitment progresses down the myeloid lineage, class II and IV HDACs appear to have more specialized roles.

### Do HDACs contribute to primitive hematopoiesis and the maintenance of tissue macrophage populations under homeostasis?

Not surprisingly, little is known about how HDACs contribute to the establishment of resident tissue macrophages from embryonic precursors. Non-mammalian models have recently been used to test how HDAC activity may regulate primitive hematopoiesis. Zebrafish embryos treated with VPA failed to initiate primitive hematopoiesis because of a block in expression of critical hematopoietic transcription factors such as Scl, Lmo2 and Gata2.^36^ Conversely, ectopic expression of human HDAC1 in these embryos enhanced expression of these hematopoietic markers, suggesting that one or more class I HDACs is required for primitive hematopoiesis. Primitive hematopoiesis in *Xenopus* occurs in analogous compartments to that of zebrafish; erythropoiesis occurs in posterior ventral blood islands and myelopoiesis in the anterior ventral blood islands. Shah *et al.*^37^ demonstrated that either VPA or trichostatin A (TSA) treatment during gastrulation resulted in the absence of erythrocytes, but did not affect the myeloid lineage. The same study also examined isolated yolk-sacs from mouse embryos at E7.5; similar to the findings with *Xenopus*, VPA caused a dose-dependent loss of erythroid progenitors without affecting overall cell viability. Using more selective inhibitors, the authors also identified class I HDACs as having important roles in the first wave of blood cell development. Collectively, these data suggest that HDACs have more pronounced roles in erythroid development during primitive hematopoiesis, but more sophisticated mechanistic studies are now required to ascertain whether specific HDACs regulate embryonic macrophage development and whether this impacts on adult tissue-resident macrophage populations. If so, the ontogeny of a given tissue-resident macrophage population may be an important variable in how specific macrophage populations are affected by HDACi.

## HDACs in the control of monocyte/macrophage activation

### HDAC-mediated regulation of innate immune pro-inflammatory signaling pathways

**Figure 1:**
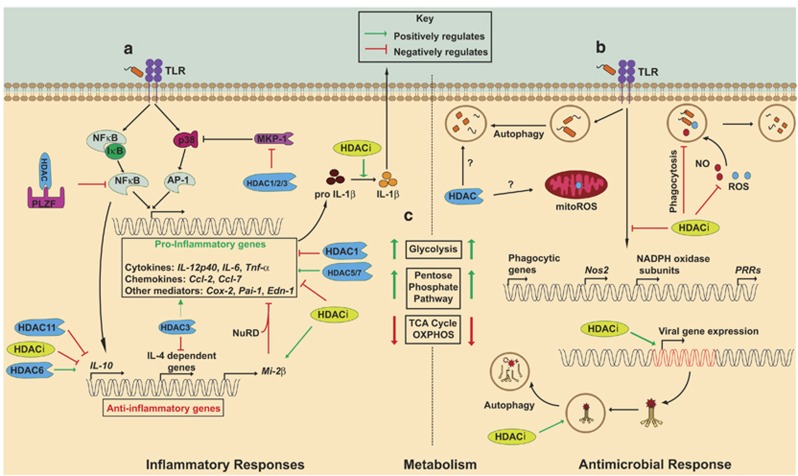
HDACs in macrophage inflammatory, antimicrobial and metabolic pathways. (**a**) TLR signaling pathways can be both positively (for example, HIF-1α and MAP kinase phosphatase-1 (MKP-1)) and negatively (for example, NF-κB) regulated by HDACs. (**b**) HDACs control expression of genes required for phagocytosis, the respiratory burst and NO production. Although the roles of HDACs in TLR-inducible autophagy and mitochondrial ROS (mitoROS) production in macrophages are still emerging, HDACi have been shown to promote autophagy-mediated clearance of HIV-1 from macrophages. (**c**) Glycolysis and the TCA cycle are intimately linked with macrophage activation status. Correlations between metabolic pathways and macrophage inflammatory and antimicrobial responses are shown. Effects of HDACi are denoted in light green, whereas individual HDACs are shown in blue. Positive regulation is depicted with a green arrow, whereas negative regulation is shown in red.

Monocytes, inflammatory macrophages and tissue-resident macrophages have an inherent plasticity, enabling them to shape and coordinate inflammatory responses, microbial destruction, tissue repair and wound healing. Indeed, a broad spectrum of macrophage phenotypes can exist, depending on the specific microenvironment and stimuli encountered.^38^ Pattern recognition receptors lie at the heart of most inflammatory processes, and thus pattern recognition receptor signaling pathways, and macrophages in general, represent tractable targets for inflammation-related diseases. The efficacy of HDACi in numerous animal models of inflammatory diseases has sparked great interest in HDACs as inflammation targets (reviewed in Shakespear *et al.*^7^ and Halili *et al.*^8^). Specific anti-inflammatory mechanisms of HDACi, as well as how individual HDAC enzymes promote innate immune pro-inflammatory responses, have been identified in recent years (Figure 1). Many such studies have focused on TLR and type I IFN signaling pathways. However, it is clear that HDACi can also have pro-inflammatory effects in certain settings, and that specific HDACs constrain inflammatory signaling (Figure 1). Indeed, it seems that an individual HDAC enzyme can have both pro- and anti-inflammatory functions in different molecular pathways, thus highlighting some of the challenges associated with selective HDAC targeting. We have previously reviewed this area in detail,^7^ and below we highlight key concepts and recent developments in this area.

### Anti-inflammatory effects of HDACi and pro-inflammatory HDAC functions

Some of the earlier reports on the anti-inflammatory properties of HDACi highlighted that the broad-spectrum HDACi, suberoylanilide hydroxamic acid (SAHA)^39^ and ITF2357,^40^ diminished production of key pro-inflammatory cytokines from lipopolysaccharide (LPS)-stimulated peripheral blood mononuclear cells. Similar effects were reported in subsequent studies.^25, 41, 42^ Several mechanisms appear to contribute to these anti-inflammatory effects. HDACi impaired the recruitment of the RelA subunit of nuclear factor (NF)-κB and IFN regulatory factor-1 to the promoter of the HDAC-dependent *IL-12p40* gene in murine DC.^42^ Another mechanism relates to HDACi-triggered hyperacetylation of mitogen-activated protein kinase phosphatase-1, which leads to inhibition of LPS-induced mitogen-activated protein kinase p38 activation.^43^ In this case, the class I HDACs, HDAC1, 2 and 3 were linked to deacetylation of mitogen-activated protein kinase phosphatase-1, which enhanced TLR-inducible mitogen-activated protein kinase p38 activation. However, other studies did not observe notable effects on TLR-activated mitogen-activated protein kinase signaling pathways in different cell populations,^42, 44^ suggesting that there may be cell type-specific effects in HDACi-mediated control of mitogen-activated protein kinase phosphatase-1 activation. The anti-inflammatory effects of HDACi have also been linked to their capacity to target the pro-inflammatory transcription factor hypoxia-inducible factor (HIF)-1α.^32^ A number of classical HDACs can associate with and/or regulate HIF-1α function.^7^ Moreover, HDAC7, which is expressed at elevated levels in inflammatory macrophages, interacts with HIF-1α and promotes inducible expression of a sub-set of LPS target genes in macrophages.^32^ Intriguingly, this effect was restricted to HDAC7-u, an alternatively spliced isoform of HDAC7 lacking the first 22 amino acids at the N-terminus. The full-length HDAC7 isoform did not have this effect; this likely relates to the inability of HDAC7-u to bind the transcriptional co-repressor CTBP1, thus licensing this isoform as a transcriptional activator. Like HDAC7, HDAC5 was also shown to promote LPS-inducible inflammatory responses in human and mouse macrophage cell lines,^45^ although whether this also involves HIF-1α is unknown at this stage. Interestingly, HDAC7 exerts many of its biological effects through an interaction with HDAC3, which can provide enzymatic activity to the complex.^46^ In this respect, *Hdac3*^−/−^ macrophages also have impaired inflammatory responses, which was attributed to the loss of IFN-β–STAT signaling.^47^ An independent study also reported that *Hdac3*^−/−^ macrophages are polarized toward an alternatively activated phenotype, as evidenced by expression of associated markers such as *Clec7a* and *Arg1*.^29^ HDAC3 binds to the enhancer regions of PU.1 target genes and suppresses IL-4-dependent gene expression. Thus, loss of HDAC3 releases this break and polarizes the macrophages to an alternatively activated phenotype. Finally, another broad mechanism by which HDACi can exert anti-inflammatory effects is through upregulating the expression or activity of anti-inflammatory molecules or pathways, as has been reported for the Mi-2/NuRD transcriptional repressor complex.^41^ This may also be the case for IL-10, as HDAC11 suppresses the LPS-inducible expression of this anti-inflammatory cytokine.^48^

### Pro-inflammatory effects of HDACi and anti-inflammatory HDAC functions

Not surprisingly, given the broad effects of HDACi in regulating diverse cellular processes, HDACi can also amplify specific macrophage inflammatory responses. For example, TSA enhanced LPS-inducible expression of *Cox-2*^25^ and *Pai-1*^49^ in macrophages. Similarly, the HDACi, LAQ824, also amplified certain LPS responses in murine macrophages.^50^ In terms of mechanisms by which specific HDACs suppress macrophage inflammatory responses, most studies point toward the involvement of class I HDACs. For example, in addition to promoting inflammatory responses in macrophages (above), the class I HDAC, HDAC3, can limit NF-κB-dependent inflammatory gene expression, as part of a complex containing the promyelocytic leukemia zinc-finger protein.^51^ In addition to HDAC3, HDAC1 acts as a negative regulator of TLR signaling by targeting the promoters of inflammatory genes such as *Il-12p40*, *Cox-2* and *Ifnβ* (reviewed in Shakespear *et al.*^7^), probably acting, at least in part, by associating with the p65 subunit of NF-κB.^52^ HDACs appear to be particularly important for feedback control of TLR-activated inflammatory responses.^25^ A recent study identified one specific mechanism by which this can occur; the ten eleven translocation family member Tet2 is LPS-inducible in macrophages and recruits the class I HDAC, HDAC2, to the *Il-6* gene to switch off the expression of this pro-inflammatory cytokine.^53^ Pro-inflammatory effects of HDACi may also relate to the capacity of some HDACs to promote anti-inflammatory responses. For example, in contrast to HDAC11, HDAC6 acts as a transcriptional activator of IL-10.^54^ HDAC6 was recruited to the *Il-10* promoter, and genetic knockout or knockdown of HDAC6, as well as inhibition of its enzymatic activity, diminished the levels of IL-10 produced by murine macrophages. Finally, as an example of amplification of inflammatory responses independently of classical gene regulation, HDACi amplified LPS-induced secretion of IL-1β in human and murine primary DC and macrophages, without altering the expression of *pro-IL-1β*.^55^ In this case, HDACi triggered inflammasome-independent IL-1β cleavage and release. Given the central role of this cytokine in inflammation-associated pathology, this finding again highlights potential contraindications that may arise from the use of pan HDACi.

### HDAC functions in macrophage-mediated host defense

Monocytes and macrophages have several roles in host defense. Through their danger detection systems, they initiate and coordinate inflammatory responses to limit the spread of infection.^56^ They also act as professional phagocytes, targeting ingested microorganisms for immediate destruction, via reactive oxygen species (ROS) and the degradative environment of the phagolysosome.^56^ Finally, TLR signaling targets persistent intracellular pathogens by upregulating the expression of genes involved in direct antimicrobial responses. For example, TLR-inducible expression of inducible nitric oxide (NO) synthase, encoded by *Nos2*, enables mouse macrophages to generate NO as an alternative means of free radical attack against intramacrophage pathogens such as *Mycobacterium* and *Leishmania*.^57^ HDACi have been shown to impair innate immune responses to infection, both *in vitro* and *in vivo,* in mouse models,^41, 58^ thus implying that HDACs have essential roles in host defense. Below, we focus on HDAC-mediated control of macrophage antimicrobial responses.

Phagocytosis requires the recognition of an external particle by either opsonic or non-opsonic receptors. Transcriptome analysis of TSA-treated murine bone marrow-derived macrophages revealed widespread inhibition of TLR-inducible expression of pattern recognition receptors, scavenger receptors and cytosolic danger-sensing receptors.^41^ TSA and VPA also downregulated the expression of other integrin receptors that are involved in the ingestion of opsonin-coated particles (for example, *Itgax*, *Itgb2*) in mouse bone marrow-derived macrophages.^58^ Consistent with such effects, pre-treatment of bone marrow-derived macrophages with HDACi impaired phagocytoic uptake of *Escherichia coli* and *Staphylococcus aureus*. Furthermore, in *in vivo* infection models in mice, HDACi increased susceptibility to *Klebsiella pneumoniae* and *Candida albicans*. Engagement of phagocytic receptors is intimately linked to the assembly of the NADPH oxidase system and the immediate generation of ROS upon particle uptake, a process known as the respiratory burst. Pre-treatment of bone marrow-derived macrophages with HDACi also disrupted the respiratory burst; total cellular ROS production was inhibited upon microbial challenge or triggering with phorbol myristate acetate.^58^ This was consistent with HDACi-mediated downregulation of expression of many of the genes encoding NADPH oxidase subunits. Several of these are also upregulated by IFN-γ, thus enabling this pro-inflammatory cytokine to prime the respiratory burst in macrophages. Treatment of DC with the HDACi, LAQ824, led to a selective defect in IFN-γ production from Th1 cells,^59^ providing an additional mechanism by which HDACi may compromise NADPH oxidase-dependent ROS production by macrophages.

TLR-inducible antimicrobial responses in macrophages include NO generation, autophagy and mitochondrial ROS production. Treatment of murine macrophages with the HDACi TSA, MS-275 or SAHA was reported to disrupt inducible NO production,^43, 60^ and HDAC1/2/3 silencing had similar effects.^43^ Similarly, the HDACi KBHA42 inhibited LPS-induced *Nos2* mRNA and NO production in RAW264.7 macrophage-like cells and peritoneal macrophages.^61^ However, HDACi did not affect NF-κB binding to response elements in the *Nos2* promoter, implicating alternative mechanisms. Instead, HDACi treatment increased recruitment of a transcriptional repressor complex, containing cyclin-dependent kinase-8, cyclin C and the mediator complex-12 and -13, at the promoter of *Nos2* gene,^60^ providing one potential mechanism. Apart from an NF-κB-binding site, the *Nos2* gene promoter harbors Oct1 and Oct2 response elements, and TSA pre-treatment also reduced LPS-inducible Oct2 expression in RAW264.7 cells.^62^ It should also be noted that some apparent inconsistencies exist in the literature regarding HDAC-regulated *Nos2* expression. For example, one study reported that, unlike TSA, other HDACi (SAHA, VPA and sodium butyrate) did not affect the production of NO from RAW264.7 cells or primary murine macrophages.^63^ Moreover, the HDACi TSA, SAHA and M344 were reported to amplify LPS-induced NO levels in microglial cells in a dose-dependent manner.^64^ It is possible that these discrepancies may relate to differences in the phenotypes of the macrophage populations being studied and/or concentrations of HDACi being used. Despite the interest in the roles of TLR-inducible autophagy and mitochondrial ROS generation in pathogen elimination, as yet, little is known of how HDACs regulate these two antimicrobial programs in macrophages. Studies in other cellular systems would suggest that this is likely to be the case,^65, 66^ and indeed, HDACi reduced human immunodeficiency virus-1 (HIV-1) release from macrophages by promoting autophagosomal degradation.^67^ Further investigations of HDAC-mediated control of autophagy and mitochondrial ROS production in macrophages are thus clearly warranted.

In contrast to findings from mouse studies, a limited number of studies suggest that HDACi may actually boost human macrophage antimicrobial responses. This is perhaps not so surprising, given some known differences in human and mouse innate immunity.^68^ The HDACi MS-275 and butyric acid restored *HLA-DR* gene expression in *Mycobacterium*-infected human macrophage-like THP-1 cells,^69^ which would be predicted to have host-protective effects. Another study showed that pharmacological or genetic silencing of HDAC1 decreased intracellular bacterial loads of the Rickettsial pathogen *Anaplasma phagocytophilum* in infected THP-1 cells.^70^ In that report, HDAC1 bound to promoter regions of various host defense genes, presumably repressing their expression. Such studies hint that some pathogens may manipulate host HDAC machinery as part of their subversion strategies. In keeping with this view, HIV latency has also been linked to repression of HIV long terminal repeat promoter activity by class I HDACs,^71^ a phenomenon that could potentially be exploited by reactivating viral reservoirs for CD8^+ve^ T-cell-mediated clearance. Despite the clinical interest in HDACi in this particular context, there still remains surprisingly little known about HDAC-mediated control of human macrophage antimicrobial pathways, however, this will undoubtedly be an area of intense future investigation.

## Myeloid HDACs in immunometabolism

### Immunometabolism in macrophage polarization and effector functions

Under homeostatic conditions, most cells utilize the cytosolic glycolysis machinery to convert glucose to pyruvate, which is then transported to the mitochondria to enter the tricarboxylic acid cycle (TCA) as acetyl-CoA.^4^ Acetyl-CoA is then consumed by the energy-efficient, but relatively slow, process of OXPHOS to generate ATP.^4, 72^ When under stress, however, macrophages reprogram their metabolism to accumulate key carbon metabolites that have important signaling roles and/or contribute to direct antimicrobial functions.^72, 73^ Below, we provide examples of macrophage metabolic adaptations and speculate on their likely links with macrophage-expressed HDACs.

The ability of myeloid cells to divide, differentiate and carry out effector functions in response to sterile and infectious stimuli requires changes in their metabolic state to enable available nutrients to be directed into distinct cellular metabolic pathways such as glycolysis, the pentose phosphate pathway (PPP) and the TCA cycle.^72, 73^ Resting macrophages generally utilize the TCA cycle to generate ATP via OXPHOS.^72^ However, the balance between OXPHOS and glycolysis is shifted in TLR-activated macrophages, with a decrease in OXPHOS and an increase in glycolysis that is accompanied by enhanced pyruvate to lactate conversion.^72, 74^ The PPP, which is critical for NADPH-dependent ROS production, is also highly active in classically activated macrophages.^72, 74^ On the other hand, alternatively activated macrophages display reduced lactate production and secretion, instead utilizing fatty acid oxidation, an intact TCA cycle and OXPHOS as hallmark metabolic features.^72^ Recent metabolic profiling of macrophages in different activation states has shed insights into mechanisms responsible for such effects. For example, in IFN-γ+LPS-activated macrophages, the TCA cycle is interrupted at the point of conversion of isocitrate to α-ketoglutarate, probably because of rapid downregulation of isocitrate dehydrogenase 1 that catalyzes this reaction.^75^ Similarly, another study showed that LPS-mediated downregulation of carbohydrate kinase-like protein in macrophages was required for metabolic reprogramming and for optimal pro-inflammatory signaling and cytokine responses.^74^ Such findings suggest that targeting specific metabolic pathways may enable manipulation of macrophage activation and effector functions. Indeed, inhibiting glycolysis attenuated LPS-inducible *pro-IL-1β* expression in LPS-activated macrophages.^3^ Interestingly, some of the metabolites that accumulate in activated macrophages can contribute to macrophage effector functions by acting as direct antimicrobial agents (for example, itaconic acid) and/or signaling molecules. In the case of the latter, the TCA cycle intermediate succinate has been linked to stabilization of HIF-1α and sustained pro-IL1β expression.^3^ Although key enzymes and metabolites that control both macrophage metabolism and functional status are beginning to be elucidated, there is much still to learn about the upstream signaling processes. Given their dual roles in regulating metabolism and immune functions,^76^ HDACs represent prime candidates.

### HDACs in immunometabolism

**Figure 2:**
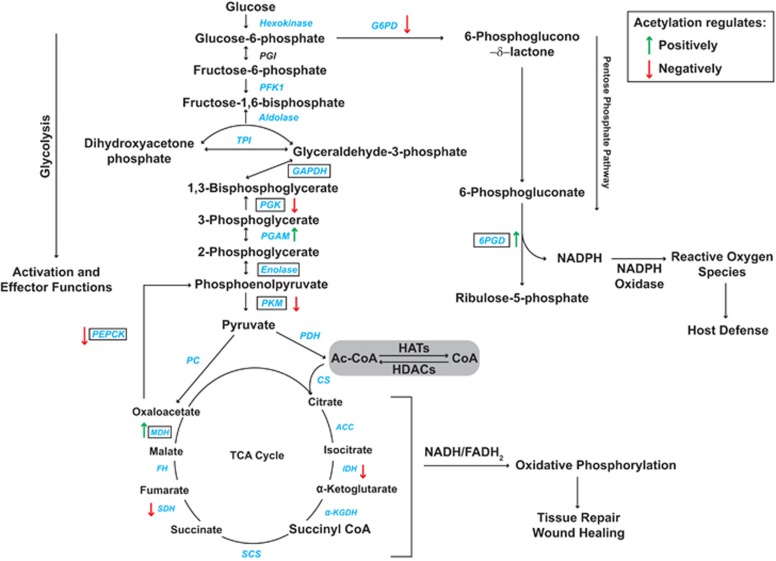
Lysine acetylation is a key control point in intermediate metabolism. Enzymes in glycolysis, the TCA cycle and the PPP for which there is evidence of acetylation are indicated in blue. Enzymes for which there is evidence of deacetylation by classical HDACs (on the basis of pharmacological inhibitors and/or functional studies with individual HDACs) are boxed, although many of these enzymes can also be deacetylated by sirtuins. Where there is clear evidence for enzyme acetylation affecting protein function in a specific way, this is indicated (green arrows: positive regulation; red arrows: negative regulation). However, in some cases, acetylation can have multiple effects on the functions of a specific metabolic enzyme.

Lysine acetylation by histone acetyl transferases involves the transfer of an acetyl group from acetyl-CoA to the target lysine residue of a protein, whereas HDACs catalyze the reverse reaction.^77^ It thus follows that histone acetyl transferases and HDACs can regulate cellular availability of acetyl-CoA,^76, 77^ a central intermediate within multiple metabolic pathways including glycolysis, OXPHOS and β-oxidation of fatty acids.^4^ It is also clear that lysine acetylation regulates the activity and functions of many enzymes in intermediary metabolic pathways, including glycolysis and the TCA cycle (Figure 2),^78, 79^ for example, by regulating enzymatic activity and/or protein stability.^77^ These observations suggest a complex regulatory loop in which intracellular concentrations of acetyl-CoA control global lysine acetylation patterns, which can in turn fine tune metabolic pathways to provide metabolic flexibility to the cell during stress.^4, 76^ Thus, histone acetyl transferases and HDACs are poised to act as molecular links between cellular metabolism, inflammation and infection.

The NAD-dependent sirtuins (class III HDACs) are thought to have particularly important roles in controlling acetylation status of various metabolic enzymes,^80^ however, classical HDACs are also likely relevant.^77, 81^ For example, TSA, a pan-inhibitor of classical HDACs, enhanced both glycolysis and OXPHOS in IFN-γ+LPS-activated macrophages,^82^ thus implicating this class of HDAC in regulating macrophage metabolic status. The surprising fact that HDACi boosted activities of both of these metabolic pathways in activated macrophages suggests that classical HDACs may regulate both cytoplasmic and mitochondrial metabolic enzymes. Further evidence of the interplay between HDACs and metabolic pathways in macrophages comes from the observation that LPS promotes the degradation of HDAC4 in macrophages, and this process requires active glycolysis.^33^ Detailed acetylome analyses have not yet been reported in myeloid cells, and little is known about how acetylation status of metabolic enzymes affects macrophage metabolism, phenotypes and effector functions. Nonetheless, studies in other cellular systems suggest that this is very likely to be the case.

Glyceraldehyde-3-phosphate dehydrogenase (GAPDH) catalyzes the conversion of glyceraldehyde-3-phosphate to 1,3 bisphosphoglycerate (step 6) in the glycolysis pathway. In mammalian cells, glucose-induced acetylation of GAPDH at K254 enhanced its enzymatic activity, whereas glucose deprivation promoted an interaction with HDAC5, its deacetylation and a reduction in activity.^83^ Thus, deacetylation of GAPDH by classical HDACs would be predicted to limit glycolysis in macrophages, perhaps leading to a corresponding reduction in inflammatory responses. Interestingly, like many metabolic enzymes, GAPDH also has ‘moonlighting functions' for example, it can translocate to the nucleus to regulate gene expression. Under apoptotic stress, acetylation of residues K117, 227 and 251 is required for the nuclear translocation of GAPDH.^84^ Thus, GAPDH acetylation may be an important switch controlling metabolic and gene regulatory functions. Another enzyme in the glycolysis pathway, phosphoglycerate mutase 1, catalyzes the interconversion of 3-phosphoglycerate to 2-phosphoglycerate (step 8). Acetylation of lysine residues K251, K253 and K254 changes the confirmation of this enzyme, enhancing its activity.^77^ Glucose deprivation results in deacetylation of phosphoglycerate mutase 1, reducing its activity and causing a switch from glycolysis to fatty acid oxidation.^85^ As glucose uptake and glycolysis is enhanced in activated macrophages, deacetylation of phosphoglycerate mutase 1 may provide an additional regulatory mechanism to control glycolysis and the inflammatory status of macrophages.

Parallel to glycolysis, the PPP has a specialized role in innate immune cells in generating reducing power for the respiratory burst, and there is some evidence that classical HDACs regulate key enzymes in this pathway. For example, in cancer cells, HDAC4 deacetylates 6-phosphogluconate dehydrogenase at residues K76 and K294, resulting in decreased enzymatic activity and inhibition of glucose metabolism via the PPP shunt.^86^ Thus, deacetylation of 6-phosphogluconate dehydrogenase by classical HDACs in macrophages may provide a regulatory mechanism constraining the PPP and macrophage antimicrobial responses. As one example of acetylation-dependent control of the TCA cycle, enzymatic activity of malate dehydrogenase, which catalyzes the conversion of malate into oxaloacetate,^78^ is enhanced by acetylation at residues K185, 301, 307 and 314.^78^ Both glucose and the pan-HDAC inhibitor TSA acetylated malate dehydrogenase to increase its activity in human liver cells.^78^ Thus, HDAC-mediated deacetylation of malate dehydrogenase would be predicted to impair the TCA cycle. It seems unlikely that this relates to fragmentation of the TCA cycle in LPS-activated macrophages as this has been specifically linked to impaired isocitrate dehydrogenase 1 activity,^75^ but it remains possible that deacetylation-mediated inhibition of malate dehydrogenase provides a means of fine tuning the TCA cycle in macrophages responding to other stimuli. Moreover, given that virtually all of the TCA cycle enzymes can be acetylated (Figure 2),^78^ acetylation-dependent control of OXPHOS is likely to be operational in macrophages. This conclusion is supported by the observation that pan-HDAC inhibition enhanced OXPHOS activity in activated macrophages.^82^ Further studies on the roles of specific classical HDACs in regulating metabolic pathways in macrophages are now needed to generate new insights into the control of inflammatory and antimicrobial responses in these cells.

## Manipulating macrophage functions for therapeutic benefit with HDACi

HDACi such as Vorinostat/SAHA have been used clinically as chemotherapeutic agents because of their ability to arrest cell cycle, induce apoptosis, promote cell differentiation and shape antitumor immune responses.^87^ Despite their clinical use, pan HDACi have also been linked to a number of adverse effects. These include gastrointestinal pathophysiologies (nausea, vomiting and anorexia), hematologic (thrombocytopenia and anemia) and metabolic (liver toxicities) complications,^88^ as well as increased expression of drug efflux systems,^9^ and pathways that can promote tumor survival and/or immune escape mechanisms,^9^ all of which may compromise their effectiveness and/or utility. On the basis of some clinical studies,^88^ it has been proposed that chemotherapy with HDAC inhibitors may increase susceptibility to infectious diseases. However, such reports can be difficult to interpret, given the immunocompromised state of late-stage cancer patients. Moreover, treatment of systemic-onset juvenile idiopathic arthritis patients with the HDAC inhibitor givinostat for 12 weeks was not associated with increased risk of infection.^89^ Nonetheless, there are clearly concerns about potential long-term side effects of HDACi therapeutic regimes. For example, a clinical trial assessing the efficacy of vorinostat in reactivating HIV transcription in patients on suppressive antiretroviral therapy noted profound effects on gene expression at day 84 of the trial, which was 70 days post-cessation of vorinostat treatment.^90^ Interestingly, gene expression changes at this long time point were apparent in T cells, but were most pronounced in monocytes and DC. Such effects could potentially contribute to variable efficacy of HDACi in reactivating HIV transcription,^91^ but also highlight concerns about enduring effects of HDACi that may be maintained long after treatment for a particular condition has been completed. These various issues relating to pan HDACi have driven the pursuit of selective HDACi for anti-inflammatory applications, and there has also been some interest in the possibility of selectively targeting pan HDACi to macrophages.

HDAC-selective inhibitor development poses a number of significant challenges. Bantscheff *et al.*^92^ showed that the inhibitory effects of various HDAC inhibitors depend upon the state of the HDAC in question. For instance, the HDACi, BML-210 inhibited recombinant HDAC1, HDAC2 and HDAC3, but failed to inhibit the HDAC-Sin3A complex, which contains HDAC1 and HDAC2. In contrast, the NCoR-HDAC3 complex was effectively inhibited at low micromolar concentrations. Thus, it is not at all clear that inhibition of individual HDACs in enzyme assays will translate to inhibition of the same HDACs in cells. Generally, it is considered that class IIa HDACs are catalytically inactive, and that they can only deacetylate target proteins by virtue of their association with HDAC3.^93^ However, this assumption is typically based on enzyme assays using acetylated peptides derived from histone proteins as HDAC substrates, and it is now clear that class IIa HDAC enzymes are active enzymes.^94^ Furthermore, class IIa HDACs can function as acetyl-lysine binding proteins, and thus class IIa HDACi also have the potential to antagonize this function. The development of class IIa-selective HDACi has generally lagged by comparison with class I- and class IIb-selective HDACi, although some have been reported.^95^ Interestingly, this study showed that class IIa HDACi have more pronounced effects on myeloid gene expression and functions by comparison with other cell types such as lymphocytes. Thus, class IIa HDAC-selective inhibitors may have applications for manipulating macrophage functions, though the literature reviewed above demonstrates that several other HDACs likely represent viable macrophage targets in different contexts.

Even compounds with high specificity for a particular protein target can have undesirable side effects that stem from that target having different functions in different cell types. This is of particular concern for targeting HDACs, as many members of this family are widely expressed across different cell types. Thus, even a highly selective inhibitor against a single HDAC enzyme may trigger unwanted biological effects. An attractive approach that could potentially address this roadblock is to target small molecules selectively to macrophages. The unique phagocytic capacity of macrophages provides one avenue toward this goal.^96^ Others include use of macrophage-expressed receptors such as folate receptors for drug uptake and delivery,^97, 98^ and the use of pro-drugs that can be hydrolyzed by macrophage-expressed enzymes to concentrate active drug within these cells. With respect to the latter strategy, the serine hydrolase, carboxyesterase-1 is highly enriched in human macrophages and hepatocytes, and a previous study showed that a specific pro-drug could be cleaved to release the broad-spectrum HDACi SAHA within the macrophage cytosol, resulting in greatly enhanced activity in these cells, by comparison with other cell types.^99^ Moreover, the same report showed that a macrophage-targeted HDACi had increased efficacy, by comparison with the parent compound, in a mouse arthritis model. More recently, clinical trials in cancer have been instigated using a compound derived from this original study.^100^ Given the interest in potential clinical applications of HDACi in clearing latent HIV reservoirs, the recent demonstration that HDACi also promote viral clearance from macrophages^67^ is likely to generate further interest in such approaches. Moreover, this finding may also be applicable to other intramacrophage pathogens. Macrophage-targeted HDACi may also offer another advantage in avoiding potential complications associated with reactivation of latent viral infections in other cell types. In a pilot study investigating the safety and efficacy of the HDACi romidepsin for patients with extranodal natural killer/T-cell lymphoma, reactivation of Epstein–Barr virus was reported.^101^ Viral reactivation in these patients was associated with fever and liver damage. Others have also noted associations between HDACi therapy and reactivation of latent DNA viruses.^102^ Macrophage-targeted HDACi would be predicted to be less likely to reactivate pools of latent DNA viruses, which could be particularly advantageous when contemplating therapeutic approaches for immune-compromised patients. Beyond infectious diseases, there is clearly great interest in HDACs as inflammation targets. HDACs that modulate metabolic pathways may be of particular interest here, and one can envisage that, in the future, it may be possible to selectively deliver class I, class IIa and/or class IIb HDAC-selective inhibitors to macrophages to more specifically manipulate metabolic pathways and inflammatory responses in this cell type.

## Conclusions

The plethora of biological pathways regulated by HDACs in macrophages provides both opportunities and challenges. Based on their roles in myeloid development, macrophage inflammatory pathways, macrophage antimicrobial responses and/or macrophage metabolism, one could probably mount a case for targeting any single macrophage-expressed classical HDAC in the context of inflammation-associated diseases and/or infectious diseases. As one example, the HDAC6 inhibitor tubastatin A reduced levels of pro-inflammatory mediators and also promoted bacterial clearance, and thus survival, in a murine cecal ligation puncture models of sepsis.^103^ A pharmacological agent with the capacity to simultaneously suppress pathological inflammatory responses and boost host defense pathways may be considered as the holy grail of drug discovery in innate immunity research. However, this is tempered by the fact that even specific HDACi, including HDAC6 inhibitors, are likely to have many unwanted side effects. Tools that enable HDACi to be specifically delivered to macrophages may help to overcome such concerns, particularly in the context of isozyme-specific HDACi. As we further unravel the links between individual HDACs and metabolic, inflammatory and antimicrobial pathways in macrophages, new opportunities for therapeutic applications of HDACi in these contexts are likely to emerge in the future.
